# Global Cognition Modified the Longitudinal Relationship Between Anemia and Depression in Old Age: The IMIAS Study

**DOI:** 10.1093/geroni/igaa057.546

**Published:** 2020-12-16

**Authors:** Tamer Ahmed, Helen-Maria Vasiliadis

**Affiliations:** 1 University of Sherbrooke, Montreal, Quebec, Canada; 2 University of Sherbrooke, Sherbrooke, Quebec, Canada

## Abstract

Background: We examined the longitudinal relationships between hemoglobin concentrations or the severity of anemia and depression and whether baseline cognitive function modifies these longitudinal relationships over 4 years of follow-up. Methods: A total of 1608 community-dwelling older adults from the International Mobility in Aging Study (IMIAS) aged 65 to 74 years were recruited in Natal (Brazil), Manizales (Colombia), Kingston (Ontario, Canada), and Saint-Hyacinthe (Quebec, Canada). The study outcome was depression, defined by a score of 16 or over in the Center for Epidemiologic Studies Depression Scale (CES-D). Longitudinal associations over four years follow-up were examined using generalized estimating equations. Models reported were either unadjusted and adjusted for research sites, alcohol drinking status, body mass index, chronic conditions, activities of daily life disabilities, and polypharmacy. Results: Longuitinal relationships suggested an evidence of multiplicative interaction by baseline global cognition in which 1g/dL increase in hemoglobin concentrations there was a significant reduction in the risk of depression with a stronger effect among participants with good cognitive function (Odds Ratio (OR)=0.85, 95% CI: 0.78-0.92) compared to those with poor cognition (OR=0.89, 95% CI: 0.80-0.97). Anemia and poor cognition at baseline were associated with an increased risk of depression over 4 years of follow-up (OR=5.80, 95% CI: 1.84-18.23). Global cognition was an effect modifier of the longitudinal association between the severity of anemia and depression. Conclusion: In international samples of older adults, hemoglobin concentrations, as well as the severity of anemia, were independent risk factors for depression and these associations differed by global cognitive function.

